# Preserved Acute Pulmonary Endothelial Homeostasis with Hydrogen Gas Inhalation After Neonatal Hypoxia–Ischemia Despite Increased Neutrophil Accumulation

**DOI:** 10.3390/biomedicines14081707

**Published:** 2026-07-29

**Authors:** Takayuki Yokota, Masumi Iketani, Toui Tsuchiya, Kosuke Sakamoto, Yasuhiro Nakao, Tsutomu Mitsuie, Eri Inoue, Kota Inoue, Tomoaki Kusaka, Takayuki Wakabayashi, Kosuke Koyano, Takanori Miki, Masaki Ueno, Shinji Nakamura, Takashi Kusaka

**Affiliations:** 1 Department of Pediatrics, Faculty of Medicine, Kagawa University, Miki 761-0793, Japan; yokota.takayuki@kagawa-u.ac.jp (T.Y.); kusaka.takashi@kagawa-u.ac.jp (T.K.); 2 Molecular and Cellular Metabolism, Tokyo Metropolitan Institute for Geriatrics and Gerontology, Tokyo 173-0015, Japan; 3 Department of Cardiovascular Surgery, Faculty of Medicine, Kagawa University, Miki 761-0793, Japan; 4 Medical Engineering Equipment Management Center, Kagawa University Hospital, Miki 761-0793, Japan; 5 Department of Anatomy and Neurobiology, Faculty of Medicine, Kagawa University, Miki 761-0793, Japan; 6 Department of Pathology and Host Defense, Faculty of Medicine, Kagawa University, Miki 761-0793, Japan; 7 Department of Pediatrics, Jichi Medical University, Shimotsuke 329-0498, Japan

**Keywords:** hydrogen gas, newborn piglet, neonatal asphyxia, lung neutrophil counts, respiratory function, endothelial activation, hemodynamics

## Abstract

**Background/Objectives**: Hydrogen gas (H_2_) inhalation has shown neuroprotective effects in neonatal hypoxic–ischemic models. However, its impact on pulmonary endothelial activation and respiratory function after hypoxic–ischemic insult remains unclear. This study investigated whether H_2_ inhalation augments pulmonary endothelial activation or impairs pulmonary function during the acute phase after neonatal hypoxic–ischemic injury. **Methods**: Sixteen newborn Camborough^®^ piglets within 24 h of birth were subjected to hypoxic–ischemic insult and randomized to an untreated group (HI, *n* = 8) or an H_2_-treated group (HI-H_2_, *n* = 8). The HI-H_2_ group received 2.1–2.7% H_2_ for 6 h. Pulmonary ICAM-1 and inducible nitric oxide synthase (iNOS) expression were assessed by immunofluorescence, lung neutrophils were quantified histologically, and pulmonary function was evaluated using arterial blood gases, oxygen index, and alveolar–arterial oxygen difference. The relationship between lung neutrophil counts and right ventricular cardiac output was also examined. Results: H_2_ inhalation did not increase pulmonary ICAM-1 expression or iNOS induction compared with untreated animals. Although lung neutrophil counts were significantly higher in the HI-H_2_ group, pulmonary gas exchange remained preserved, with no significant differences in arterial blood gases, oxygen index, or alveolar–arterial oxygen difference. Lung neutrophil counts were positively correlated with right ventricular cardiac output, suggesting that enhanced pulmonary perfusion may contribute to neutrophil redistribution rather than inflammatory recruitment. **Conclusions**: H_2_ inhalation preserved pulmonary endothelial homeostasis without impairing respiratory function during the acute phase after neonatal hypoxic–ischemic insult. The observed increase in lung neutrophils was not accompanied by evidence of endothelial activation or deterioration of gas exchange, supporting the acute pulmonary safety of H_2_ inhalation while suggesting that neutrophil accumulation may, at least in part, reflect hemodynamic redistribution rather than injurious inflammatory infiltration.

## 1. Introduction

Neonatal hypoxic–ischemic encephalopathy is a major cause of morbidity and mortality in newborns worldwide. Although therapeutic hypothermia has become the standard of care [1], it is not universally effective [2], and many affected neonates experience multi-organ dysfunction, including cardiovascular, renal, and pulmonary complications [3]. Among these, respiratory impairment is particularly critical, given that perinatal asphyxia can cause surfactant inactivation, pulmonary hypertension, and inflammatory cell infiltration, leading to impaired gas exchange and prolonged respiratory support [4].

In the pulmonary circulation, endothelial activation and redox imbalance play central roles in the pathophysiology following hypoxic–ischemic (HI) insult. Upregulation of intercellular adhesion molecule-1 (ICAM-1) facilitates leukocyte adhesion and transmigration [5], while inducible nitric oxide synthase (iNOS) induction contributes to excessive nitric oxide production and peroxynitrite formation, promoting nitrosative stress and endothelial dysfunction [6]. Such endothelial inflammatory activation has been implicated in the development of pulmonary vascular dysregulation and may contribute to persistent pulmonary hypertension of the newborn (PPHN) and impaired gas exchange after asphyxia [4].

Hydrogen gas (H_2_) has attracted attention as a potential therapeutic gas owing to the work of Ohsawa et al., who demonstrated its antioxidant, anti-inflammatory, and anti-apoptotic potential both in vivo and in vitro [7,8,9]. H_2_ selectively reduces hydroxyl radical (•OH) and peroxynitrite (ONOO−), suggesting a potential role in modulating redox-dependent signaling pathways. We previously reported that H_2_ inhalation following hypoxic–ischemic insult significantly improves neurological outcomes [10], enhances cerebral hemodynamics and oxygenation [11], and attenuates seizure burden [12] in newborn piglets. These findings suggest that hydrogen therapy may exert systemic protective effects beyond the brain.

However, as H_2_ is inhaled directly into the lungs, its impact on pulmonary endothelial homeostasis after HI remains unclear. While H_2_ is generally considered anti-inflammatory, it is unknown whether H_2_ inhalation might influence pulmonary endothelial activation markers such as ICAM-1 and iNOS in the acute phase after HI [13,14]. Clarifying whether H_2_ suppresses, or at least does not exacerbate, endothelial inflammatory signaling is essential for evaluating its safety in clinical translation.

In addition, in the same neonatal piglet model, we have also demonstrated that H_2_ inhalation increases right ventricular cardiac output without impairing left heart function [15], suggesting potential hemodynamic effect of H_2_. Given that pulmonary perfusion influences leukocyte trafficking, alterations in cardiac output may affect lung immune cell distribution independently of endothelial inflammatory activation.

Therefore, the primary aim of this study was to determine whether H_2_ inhalation after HI augments pulmonary endothelial activation, as assessed by ICAM-1 expression and iNOS induction, and whether it adversely affects respiratory function during the early post-insult period. We hypothesized that H_2_ would not exacerbate endothelial inflammatory signaling nor impair pulmonary gas exchange. In parallel, we examined lung neutrophil accumulation and its relationship with cardiac output to explore whether changes in immune cell distribution might reflect hemodynamic modulation rather than endothelial injury.

## 2. Materials and Methods

### 2.1. Animal Preparation

This translational study used a well-established neonatal piglet model of hypoxic–ischemic encephalopathy (HIE) to evaluate the early pulmonary and systemic effects of H_2_ inhalation. Lung inflammatory responses were assessed over a 6 h post-insult period. Sixteen newborn piglets (Camborough^®^; Daiwa Chikusan, Kagawa, Japan), including 10 males and 6 females, within 24 h of birth and weighing 1.5–2.1 kg, were used in the experiment. The study protocol was approved by the Kagawa University Animal Care and Use Committee (15070-1) and was conducted in accordance with institutional regulations and the ARRIVE guidelines.

Anesthesia was induced with 1–2% isoflurane (Forane^®^ inhalant liquid; Abbott Co., Tokyo, Japan) in air, followed by endotracheal intubation and mechanical ventilation. Umbilical venous and arterial catheters (Atom Indwelling Feeding Tubes for Infants; Atom Medical Corporation, Tokyo, Japan) were placed for fluid administration and physiological monitoring. Pancuronium bromide (0.1 mg/kg bolus followed by 0.1 mg/kg/h infusion) and fentanyl citrate (Fentanyl Injection 0.1 mg “Daiichi Sankyo”; Daiichi Sankyo Co., Ltd., Tokyo, Japan; 10 μg/kg bolus followed by 5 μg/kg/h infusion) were administered for muscle relaxation and analgesia. Electrolyte solution containing 2.7% glucose (KN3B; Otsuka Pharmaceutical Co., Tokyo, Japan) was infused at 4 mL/kg/h. Rectal temperature was maintained at 39.0 ± 0.5 °C throughout the experiment.

### 2.2. Experimental Protocol

The hypoxic–ischemic protocol used in the present study was based on our previously established neonatal piglet model, in which both amplitude-integrated electroencephalography (aEEG) and cerebral blood volume (CBV), measured by time-resolved spectroscopy, were used as physiological guides to control the severity of the hypoxic–ischemic insult.

In our previous validation study, the combined aEEG + CBV-guided protocol more efficiently produced piglets that survived the insult while developing reproducible histopathological brain injury compared with an aEEG-guided protocol alone. Specifically, the proportion of damaged surviving piglets was higher and mortality was lower in the aEEG + CBV-guided protocol, demonstrating improved control of insult severity and reproducibility of the neonatal hypoxic–ischemic encephalopathy model.

In the present study, hypoxia was induced by decreasing the inspired oxygen concentration (FiO_2_) to 4%, targeting low-amplitude EEG activity (<5 μV). The hypoxic insult lasted 30 min, during which FiO_2_ was adjusted to maintain EEG suppression while keeping heart rate >130 beats/min and mean arterial pressure >70% of baseline values. In accordance with our established protocol, the severity and duration of hypoxia were controlled to achieve survivable hypoxic–ischemic insult while avoiding excessive circulatory collapse. Resuscitation with 100% oxygen was initiated when predefined physiological thresholds were reached. FiO_2_ was subsequently titrated to maintain peripheral oxygen saturation (SpO_2_) between 95% and 98%.

### 2.3. Hydrogen Gas Administration

After the insult, piglets were randomized into two groups: the HI group (no treatment, *n* = 8) and the HI-H_2_ group (2.1–2.7% H_2_ administered via the ventilator for 6 h, *n* = 8). Hydrogen concentration was adjusted according to oxygen requirements and continuously monitored using a gas detector (TP-70D; Riken Keiki Co., Ltd., Tokyo, Japan).

### 2.4. Physiological Measurements

Arterial blood gases were measured at predefined time points. Arterial blood gas parameters (pH, PaO_2_, and PaCO_2_), oxygen index (OI), and alveolar–arterial oxygen difference (AaDO_2_) were evaluated at baseline (pre-insult), immediately before resuscitation (0 h), and at 1, 3, and 6 h post-insult. These parameters were compared between the HI and HI-H_2_ groups to assess pulmonary function over time. The oxygen index was calculated as (mean airway pressure × FiO_2_ × 100)/PaO_2_. AaDO_2_ was calculated as [(FiO_2_ × (atmospheric pressure − water vapor pressure)) − (PaCO_2_/0.8)] − PaO_2_, using standard sea-level values (atmospheric pressure: 760 mmHg; water vapor pressure: 47 mmHg).

### 2.5. Echocardiographic Assessment

Echocardiographic evaluations were performed using an Xario100S ultrasound system (Canon Medical Systems Corporation, Otawara, Japan) with a 12 MHz multifrequency sector probe. Left and right ventricular outflow velocity patterns were obtained from parasternal short-axis and long-axis views using pulsed-wave Doppler with the sample volume positioned just above the aortic or pulmonary valve. Stroke volume (SV) of both ventricles and right ventricular outflow tract velocity-time integral (RVOT-VTI) were calculated by tracing the Doppler flow profile offline. Cardiac output (CO) for each ventricle was calculated as SV multiplied by heart rate. These measurements were performed in accordance with our previously reported methodology in the same neonatal piglet hypoxic–ischemic model [15]. Echocardiographic assessment was incorporated during the later phase of the study as an additional physiological monitoring modality within the approved experimental protocol. This did not alter the total number of animals, the experimental procedures, or the predefined humane endpoints. Therefore, complete echocardiographic measurements were available only in a subset of animals (*n* = 5 per group), whereas other physiological and histological analyses were performed in all animals (*n* = 8 per group).

### 2.6. Histological and Immunofluorescence Analysis

At 6 h after the insult, piglets were euthanized under deep anesthesia. Sedation with fentanyl and muscle relaxation with pancuronium bromide were maintained intravenously. While animals remained intubated, inhalation anesthesia with isoflurane was administered via a vaporizer (5% isoflurane) until respiration ceased and death was confirmed.

The lungs were perfused with saline and fixed with 4% paraformaldehyde. Lung tissues were processed for histological analysis and immunohistochemistry using an anti-PM1 antibody (T-3503; BMA Biomedicals, Augst, Switzerland). PM1-positive cells with lobulated nuclei were counted in ten randomly selected high-power fields (400× magnification) per sample.

For immunofluorescence analysis, paraffin-embedded lung sections (5 μm thickness) were deparaffinized, rehydrated, and subjected to antigen retrieval by heating in 10 mM citrate buffer (pH 6.0) at boiling temperature for 5 min. After blocking with bovine serum albumin, sections were incubated overnight at 4 °C with primary antibodies against ICAM-1 (1:100; 10831-1-AP, Proteintech Group, Inc., Rosemont, IL, USA) and iNOS (1:100; NB300-605, Novus Biologicals, Centennial, CO, USA). After washing, appropriate fluorescence-conjugated secondary antibodies were applied. Nuclei were counterstained with Hoechst 33342 (1:1000; #1917251, Nacalai Tesque, Kyoto, Japan). Fluorescence images were acquired using a confocal laser-scanning microscope under identical exposure settings for all groups.

For quantitative immunofluorescence analysis, three fields were randomly selected from each section by an investigator blinded to the experimental groups, and all acquired images were analyzed using ImageJ software (version 1.54p; National Institutes of Health, Bethesda, MD, USA). ICAM-1 and iNOS fluorescence intensities were measured over the entire image without signal thresholding, manual region-of-interest selection, or background subtraction. Hoechst images were binarized using the same automatic thresholding algorithm for all images without manual adjustment, and the threshold-positive area was defined as the Hoechst-positive nuclear area. ICAM-1 and iNOS fluorescence intensities were normalized to the Hoechst-positive nuclear area, and the mean value of the three fields was used as the representative value for each animal. Because field selection was randomized and blinded, and the subsequent analysis involved no manual adjustment or image exclusion, observer-dependent variability was minimized; therefore, inter-observer reliability was not assessed separately.

### 2.7. Statistical Analysis

Statistical analyses were performed using GraphPad Prism version 9.3.1 (GraphPad Software, San Diego, CA, USA). Data are presented as mean ± standard deviation (SD). Two-way repeated-measures analysis of variance followed by Tukey’s post hoc test was used for comparisons between groups. A *p* value < 0.05 was considered statistically significant. No formal sample size calculation was performed because this study was exploratory in nature.

## 3. Results

### 3.1. Lung Histology and Endothelial Marker Expression

**Figure 1:**
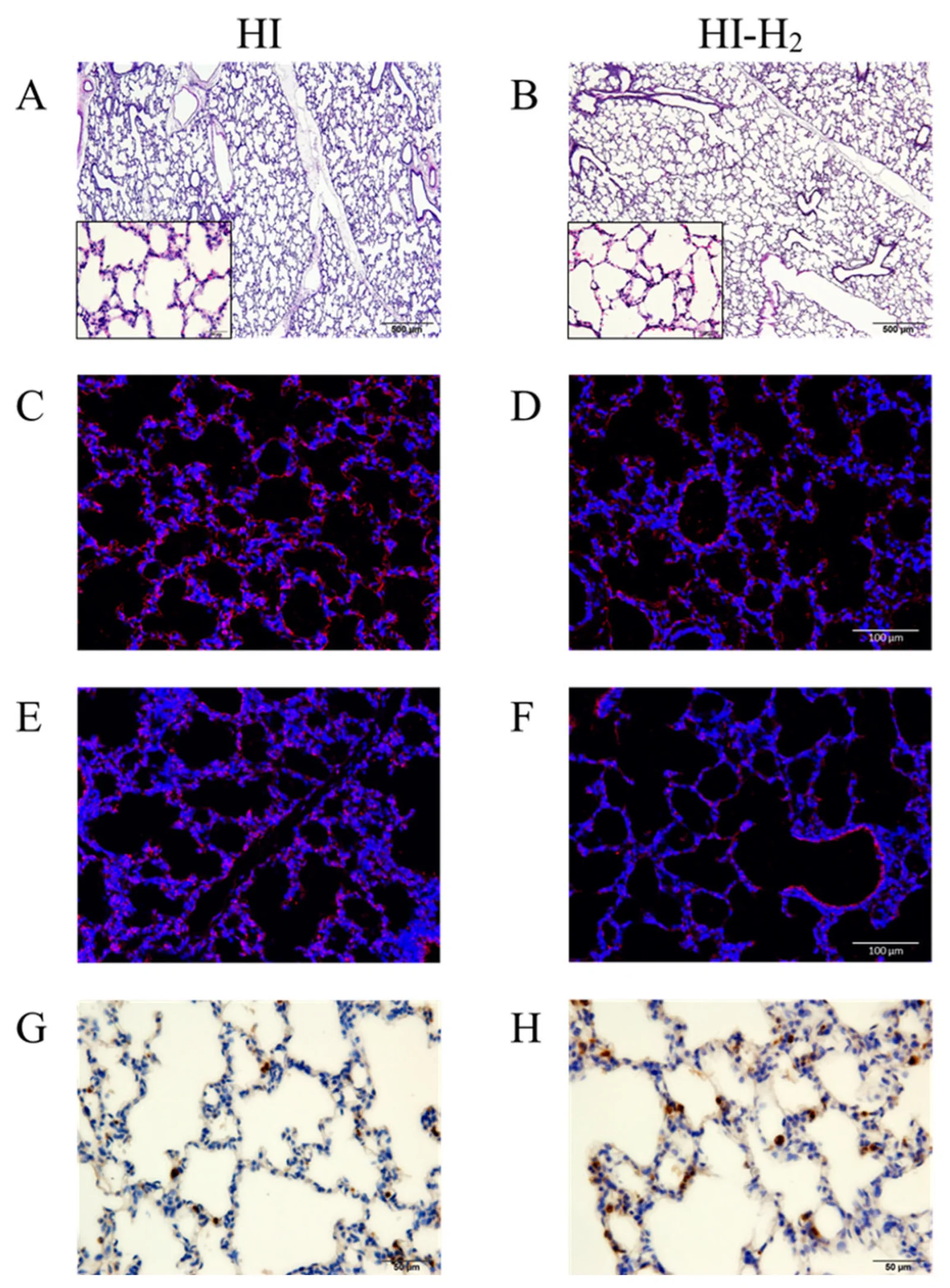
Representative lung histology and immunostaining images. (**A**,**B**) Low-magnification hematoxylin-eosin (HE)-stained lung sections from the HI (**A**) and HI-H_2_ (**B**) groups. Alveolar architecture appears preserved in both groups without overt structural disruption. Scale bar, 500 µm. (**C**–**F**) Representative immunofluorescence images of lung sections from the HI, and HI-H_2_ groups. (**C**,**D**) ICAM-1 staining. (**E**,**F**) iNOS staining. Red fluorescence indicates immunoreactivity for the target protein (ICAM-1 in (**C**,**D**); iNOS in (**E**,**F**)), and blue fluorescence indicates nuclei counterstained with Hoechst. Scale bar, 100 µm. (**G**,**H**) Representative immunostaining images using anti-PM1 antibody showing polymorphonuclear neutrophils in lung tissue from the HI (**G**) and HI-H_2_ (**H**) groups. PM1-positive cells with lobulated nuclei are visible in the alveolar spaces and interstitial regions, with a greater number observed in the HI-H_2_ group. Scale bar, 50 µm.

Representative lung histology and immunostaining images are shown in Figure 1. Hematoxylin and eosin-stained sections revealed no apparent differences in alveolar architecture between the HI and HI-H_2_ groups (Figure 1A,B).

**Figure 2:**
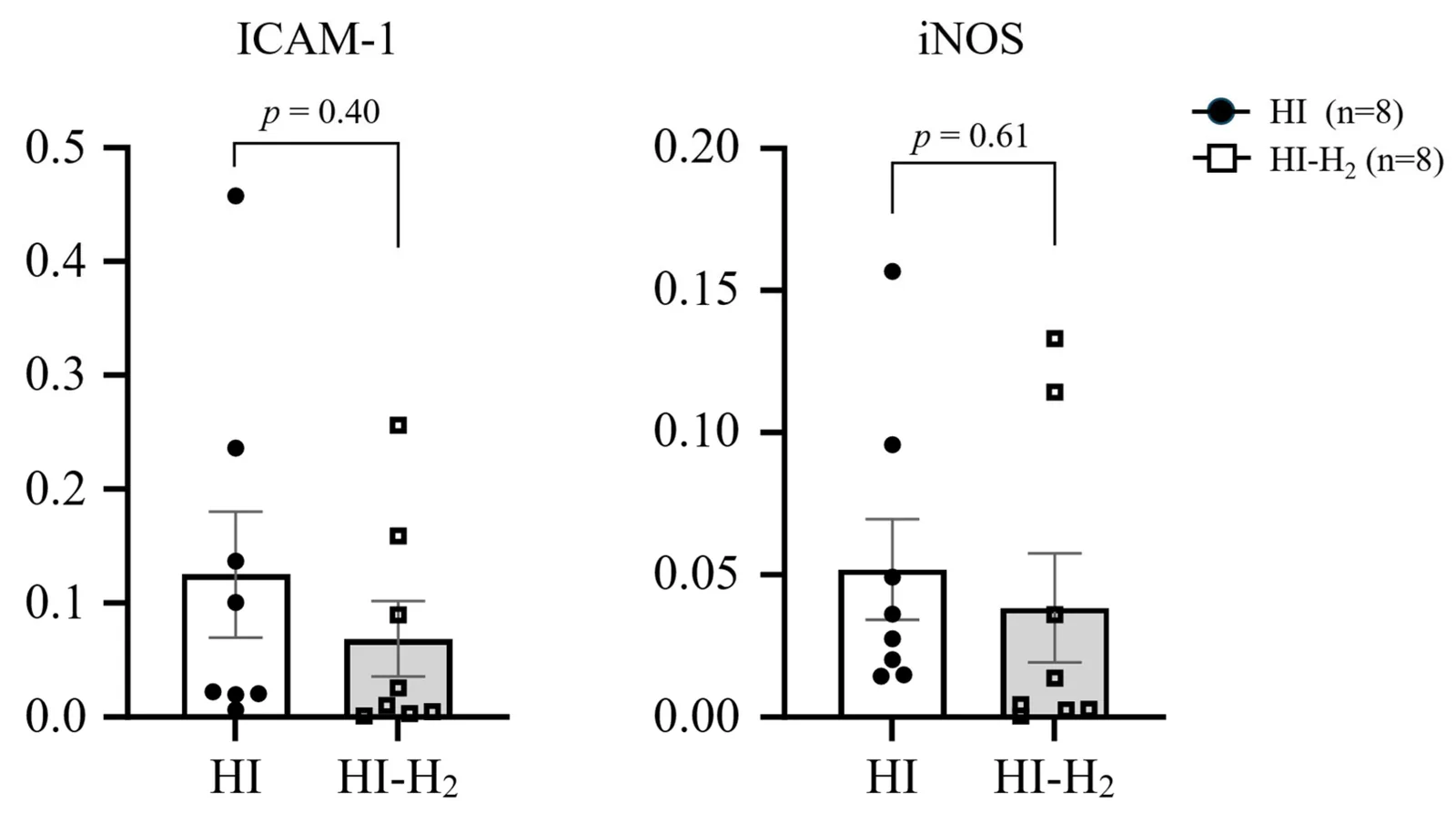
Quantification of ICAM-1 and iNOS fluorescence intensity in lung sections. Fluorescence intensity of ICAM-1 and iNOS in lung sections was quantified and normalized to the Hoechst-stained nuclear area. Data were presented as fluorescence intensity per Hoechst-positive area. No significant differences were observed between the HI and HI-H_2_ groups for either ICAM-1 or iNOS expression (ICAM-1: *p* = 0.61; iNOS: *p* = 0.40, unpaired *t*-test).

Immunofluorescence analysis demonstrated detectable ICAM-1 and iNOS expression in lung tissue following hypoxic–ischemic insult in both groups (Figure 1C–F). No apparent increase in ICAM-1 or iNOS expression was observed in the HI-H_2_ group compared with the untreated HI group (Figure 2).

### 3.2. Lung Neutrophil Quantification

**Figure 3:**
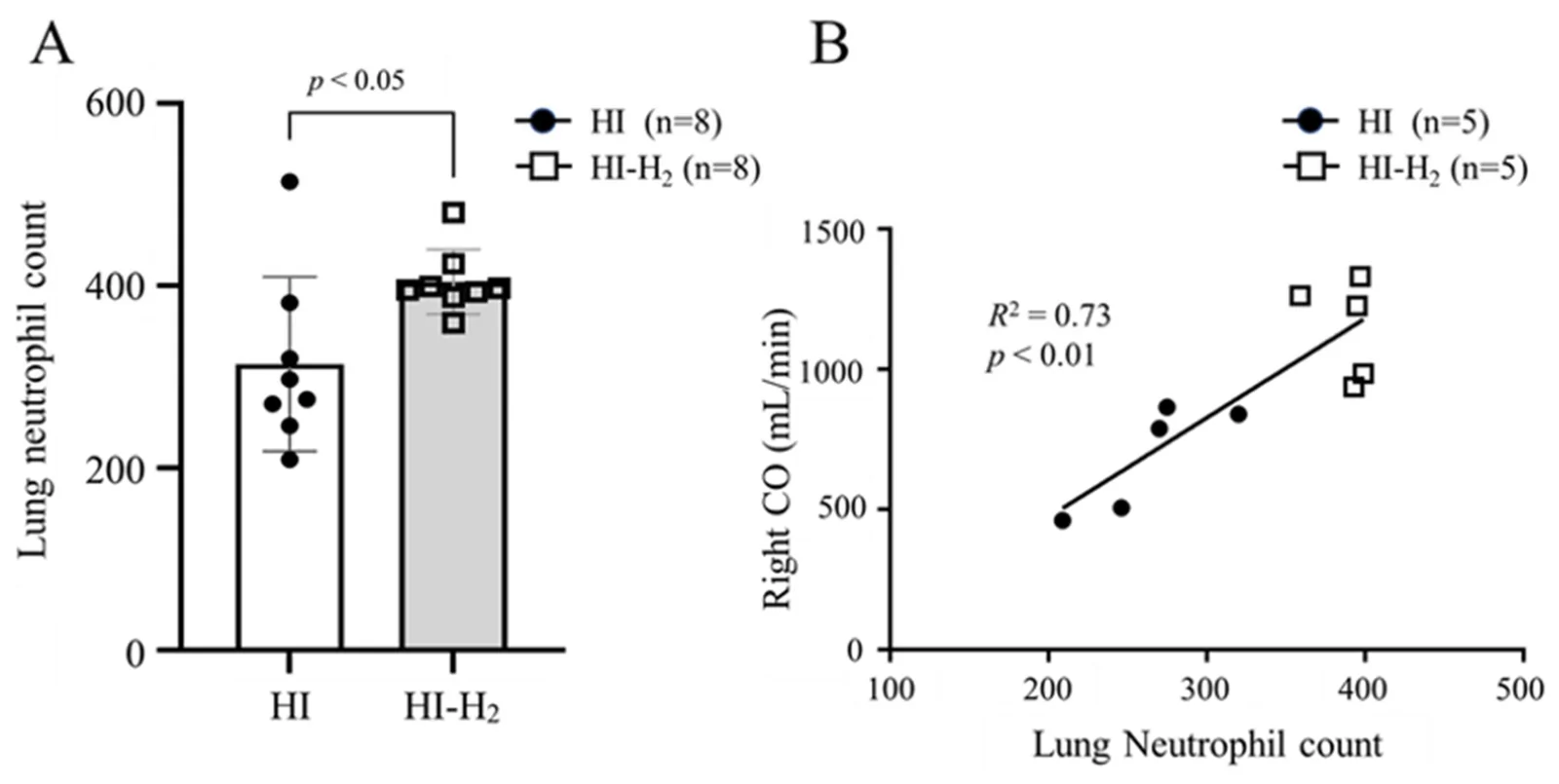
Quantitative analysis of lung neutrophils. (**A**) The number of PM1-positive neutrophils was counted in 10 randomly selected high-power fields (400×) per sample. The HI-H_2_ group demonstrated a significantly higher neutrophil count compared with the HI group (*p* < 0.05, unpaired *t*-test). (**B**) Correlation between right cardiac output (CO) and lung neutrophil counts at 6 h post-insult. A significant positive correlation (*R*^2^ = 0.73, *p* <0.01) was observed, suggesting that enhanced pulmonary perfusion may contribute to increased neutrophil presence in the lung tissue. Echocardiographic assessment was incorporated during the study as part of protocol refinement; therefore, correlation analysis was performed using animals with complete cardiac output measurements (*n* = 5 per group).

Immunostaining with anti-PM1 antibody demonstrated PM1-positive polymorphonuclear cells in the alveolar spaces and lung parenchyma of both groups. High-magnification images revealed a visually greater number of PM1-positive neutrophils in the HI-H_2_ group compared with the HI group (Figure 1G,H). Lung neutrophil infiltration was significantly greater in the HI-H_2_ group compared with the HI group at 6 h post-insult (Figure 3A; 95% CI 8.6–171.9, *p* < 0.05).

### 3.3. Pulmonary Gas Exchange

**Figure 4:**
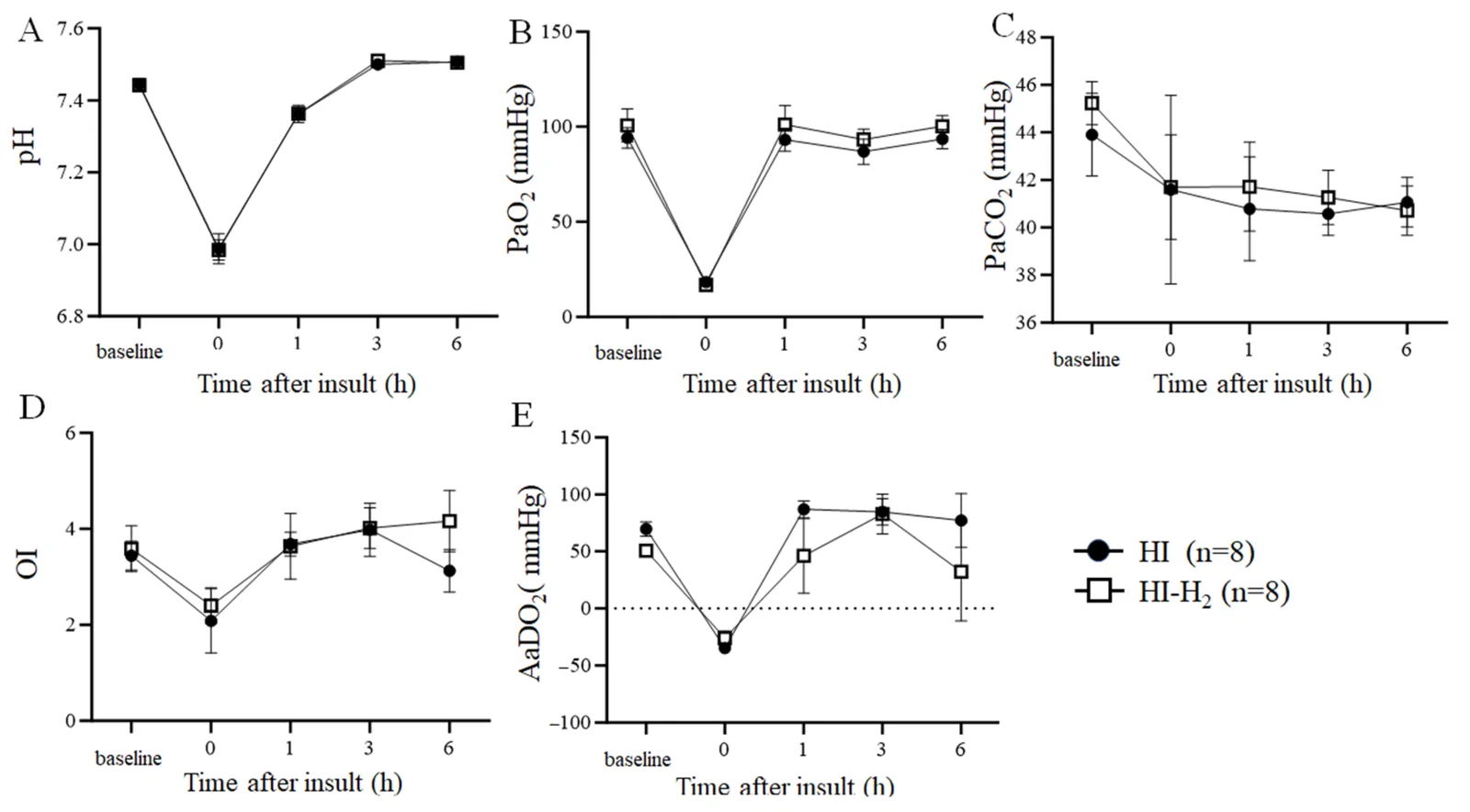
Time course of changes in arterial blood gas parameters after hypoxic–ischemic insult. The arterial blood gas parameters were measured at baseline and at 0, 1, 3, and 6 h post-insult. The five individual line graphs show changes in (**A**) pH, (**B**) PaO_2_, (**C**) PaCO_2_, (**D**) oxygen index (OI), and (**E**) alveolar–arterial oxygen difference (AaDO_2_). No significant differences were observed between the HI and HI-H_2_ groups by two-way ANOVA followed by post hoc Tukey analysis.

Despite increased neutrophil counts, no significant differences were observed in pH, PaCO_2_, PaO_2_, OI, or AaDO_2_ values between the two groups at any time point (Figure 4A–E). Both groups showed an initial drop in pH immediately after resuscitation, followed by gradual normalization. PaCO_2_ and PaO_2_ fluctuated within physiological ranges, and OI and AaDO_2_ remained stable, indicating preserved pulmonary gas exchange in both groups.

### 3.4. Cardiac Output and Hemodynamic Measurements

The left and right CO at each time point is summarized in Table 1. Echocardiographic assessment was incorporated during the study as part of protocol refinement, and thus complete cardiac output measurements were available for five animals in each group. No significant difference was observed in left CO between the HI and HI-H_2_ groups across the 6 h after insult. In contrast, right CO tended to increase in the HI-H_2_ group and reached higher values at 5 h after insult.

### 3.5. Correlation Between Right Ventricular Cardiac Output and Lung Neutrophil Counts

Notably, a strong positive correlation was found between the right CO at 5 h after insult and the number of lung neutrophil counts (Figure 3B; *R*^2^ = 0.73, 95% CI 0.50–0.97, *p* < 0.01).

## 4. Discussion

The present study demonstrated that H_2_ inhalation did not augment pulmonary endothelial activation following hypoxic–ischemic insult, as evidenced by the absence of increased ICAM-1 expression and iNOS induction. Despite this lack of endothelial inflammatory activation, lung neutrophil counts were significantly increased in the H_2_-treated group. Notably, lung neutrophil accumulation was positively correlated with right ventricular cardiac output, suggesting a potential association with increased pulmonary perfusion rather than inflammatory recruitment. Importantly, these changes occurred without impairment of pulmonary gas exchange, as reflected by stable arterial blood gas parameters, oxygen index, and alveolar–arterial oxygen difference. Taken together, these findings indicate that H_2_ inhalation preserves pulmonary endothelial homeostasis in the early phase after hypoxic–ischemic insult, while modulating immune cell distribution without inducing functional respiratory deterioration.

Endothelial activation is a key component of pulmonary pathophysiology following hypoxic–ischemic insult, characterized by upregulation of adhesion molecules such as ICAM-1 and induction of iNOS, which promotes excessive nitric oxide production and subsequent peroxynitrite formation. These processes contribute to nitrosative stress, endothelial dysfunction, and pulmonary vascular dysregulation, potentially leading to impaired gas exchange and the development of conditions such as persistent pulmonary hypertension of the newborn [4].

In the present study, H_2_ inhalation did not increase ICAM-1 expression or iNOS induction in lung tissue following hypoxic–ischemic insult. This finding suggests that H_2_ does not exacerbate endothelial inflammatory signaling or redox imbalance in the pulmonary circulation during the early post-insult phase. Given that H_2_ selectively scavenges hydroxyl radicals and peroxynitrite, it is plausible that hydrogen attenuates redox-dependent signaling pathways involved in endothelial activation, thereby preventing the amplification of inflammatory responses [16].

These results are consistent with previous reports demonstrating the antioxidative and anti-inflammatory properties of hydrogen in various models of lung injury. H_2_ has been shown to attenuate pulmonary inflammation, reduce cytokine production, and suppress oxidative stress responses in experimental models, including ventilator-induced and lipopolysaccharide-induced lung injury [13,14,17]. Furthermore, hydrogen inhalation has been reported to modulate macrophage polarization, attenuate fibrosis, and improve lung function in experimental lung injury models [18]. In addition, hydrogen has been shown to inhibit HMGB1 release via activation of the Nrf2/HO-1 pathway, further supporting its role in modulating redox-dependent inflammatory signaling [19]. Importantly, the absence of ICAM-1 and iNOS upregulation in the present study indicates that H_2_ inhalation does not promote pulmonary endothelial injury, even in the context of systemic hypoxic–ischemic stress. This supports the concept that H_2_ preserves endothelial homeostasis and may mitigate redox-driven vascular dysfunction in the neonatal lung.

In contrast to these well-established anti-inflammatory effects of hydrogen [13,14,18], the present study demonstrated increased neutrophil accumulation without evidence of endothelial activation. In general, increased neutrophil accumulation in the lung is considered a hallmark of inflammatory injury and is often associated with endothelial activation and impaired gas exchange [4,5,20,21]. However, in the present study, the increase in lung neutrophil counts occurred in the absence of upregulated ICAM-1 expression or iNOS induction, and without any deterioration in respiratory function. This dissociation suggests that neutrophil accumulation in the H_2_-treated group does not reflect inflammation-driven neutrophil recruitment mediated by endothelial activation. Previous studies have demonstrated that, in neonatal hypoxic–ischemic injury, neutrophils can accumulate within the vasculature during an early intravascular phase before significant extravasation into tissue occurs [22]. In this phase, neutrophils may remain predominantly within blood vessels and exhibit functional activity without extensive endothelial transmigration. In neonatal models, neutrophils are predominantly confined to the intravascular compartment during the early hours after injury and can contribute to tissue damage by impairing microvascular flow and oxygen delivery, even in the absence of parenchymal infiltration [23].

Instead, our findings indicate that the observed increase in lung neutrophils may be attributable to hemodynamic factors. We found a significant positive correlation between lung neutrophil counts and right ventricular cardiac output, suggesting that enhanced pulmonary perfusion may facilitate neutrophil delivery or retention within the pulmonary microvasculature. Increased blood flow may promote transient neutrophil margination or redistribution within the lung, independent of inflammatory signaling pathways [20,22,24,25].

This interpretation is consistent with the concept that leukocyte distribution within the pulmonary circulation is influenced not only by inflammatory cues but also by hemodynamic conditions. However, this interpretation remains speculative, and alternative mechanisms should also be considered. The increased neutrophil counts may reflect transient intravascular margination, altered leukocyte trafficking, or changes in endothelial–leukocyte interactions rather than true inflammatory recruitment into the lung parenchyma. Bronchoalveolar lavage (BAL) and alveolar inflammatory scoring were not performed in the present study. In addition, neutrophil activation markers, inflammatory cytokines, and direct assessment of endothelial function were not evaluated. Therefore, we cannot determine whether the increased neutrophils were predominantly sequestered within the pulmonary microvasculature or had migrated into the alveolar space. Further mechanistic studies will be required to clarify the biological significance of the observed neutrophil accumulation. The observed increase in lung neutrophils may represent, at least in part, a perfusion-driven redistribution rather than injurious inflammatory infiltration. Importantly, despite the increased neutrophil counts, pulmonary gas exchange remained preserved and endothelial inflammatory markers (ICAM-1 and iNOS) were not increased during the acute observation period, suggesting that H_2_ inhalation did not exacerbate acute lung injury. Such an uncoupling between neutrophil accumulation and endothelial inflammatory activation suggests that H_2_ inhalation may modulate pulmonary physiology without exacerbating acute lung injury [24,26].

This study has several limitations. First, the sample size was relatively small and was determined based on our previous experience with the same neonatal piglet hypoxic–ischemic model rather than on a formal a priori power calculation. Therefore, this exploratory study should be interpreted with appropriate caution, and the findings require confirmation in larger studies. Second, the evaluation period was limited to the first 6 h after hypoxic–ischemic insult. Therefore, the present findings reflect only the acute pulmonary effects of H_2_ inhalation. Later events in the inflammatory cascade, including delayed endothelial activation and changes in ICAM-1 expression or other inflammatory mediators, may not have been captured within this observation period. Accordingly, the longer-term effects of H_2_ inhalation on lung structure and function remain unknown and should be investigated in future studies with extended follow-up. Third, although we assessed endothelial activation using ICAM-1 expression and iNOS induction, we did not evaluate downstream functional consequences such as nitric oxide production, peroxynitrite formation, or other markers of oxidative and nitrosative stress. In addition, we did not directly assess neutrophil activation status, inflammatory cytokine levels, or detailed histological indicators of lung injury. Fourth, echocardiographic assessment was introduced during the later phase of the study and was therefore available only in a subset of animals (*n* = 5 per group). Consequently, the analyses of right ventricular cardiac output and its correlation with lung neutrophil counts should be regarded as exploratory. Because these measurements were not available for all animals, potential selection bias cannot be excluded, and the findings should be interpreted with caution. In addition, pulmonary arterial blood flow and pulmonary artery pressure were not directly measured. From a translational perspective, the neonatal piglet is one of the most clinically relevant large-animal models of neonatal hypoxic–ischemic encephalopathy because its cardiopulmonary physiology closely resembles that of human newborns. As H_2_ inhalation is being investigated as a potential adjunctive therapy for neonatal hypoxic–ischemic encephalopathy, demonstrating that it does not cause acute pulmonary dysfunction is an important prerequisite for clinical translation. Nevertheless, our findings are limited to the acute phase following hypoxic–ischemic insult and should not be generalized beyond this experimental model without further preclinical validation and carefully designed clinical studies.

Future studies should incorporate comprehensive redox and inflammatory profiling, including functional assessment of nitric oxide signaling and oxidative stress pathways, as well as detailed evaluation of neutrophil activation and endothelial integrity. Longer observation periods will also be necessary to evaluate delayed inflammatory responses, temporal changes in endothelial activation, and the long-term pulmonary effects of H_2_ inhalation following neonatal hypoxic–ischemic insult. In addition, direct assessment of pulmonary hemodynamics will be required to clarify the mechanisms underlying neutrophil redistribution and to further establish the pulmonary safety and therapeutic potential of H_2_.

## 5. Conclusions

In conclusion, H_2_ inhalation did not augment pulmonary endothelial activation following hypoxic–ischemic insult, as indicated by the absence of increased ICAM-1 expression and iNOS induction. These findings suggest that hydrogen did not impair pulmonary endothelial homeostasis and does not exacerbate redox-mediated inflammatory signaling in the early post-insult phase. Despite an increase in lung neutrophil accumulation, pulmonary gas exchange remained stable, and neutrophil counts were associated with increased right ventricular cardiac output. This indicates that neutrophil accumulation may, at least in part, reflect hemodynamic redistribution rather than injurious inflammatory recruitment, although the precise mechanism remains to be determined. Taken together, our results provide preclinical evidence supporting the acute pulmonary safety of H_2_ inhalation following neonatal hypoxic–ischemic insult. Although further studies with longer follow-up are required, H_2_ inhalation may represent a promising adjunctive therapy for neonatal hypoxic–ischemic encephalopathy without causing acute pulmonary endothelial injury.

## Figures and Tables

**Table 1:** Time course of changes in the left and right cardiac output (CO) after insult.

	HI Group, Mean (SD) (*n* = 5)	HI-H_2_ Group, Mean (SD) (*n* = 5)
Left CO (mL/min)		
Baseline	947 (310)	762 (245)
0 h	338 (87)	240 (113)
0.5 h	728 (278)	681 (341)
1 h	945 (218)	642 (181)
2 h	796 (161)	578 (186)
3 h	768 (232)	688 (368)
4 h	748 (167)	632 (152)
5 h	790 (292)	669 (332)
6 h	759 (340)	678 (155)
Right CO (mL/min)		
Baseline	1005 (385)	952 (227)
0 h	89 (41)	112 (78)
0.5 h	719 (294)	732 (322)
1 h	848 (252)	705 (248)
2 h	565 (218)	935 (258)
3 h	802 (164)	1019 (157)
4 h	841 (283)	1038 (139)
5 h	691 (173)	1148 (157) *
6 h	862 (317)	1075 (257)

Values are presented as mean (SD). * *p* < 0.05, versus the hypoxic–ischemic (HI) group by two-way analysis of variance, post hoc Tukey analysis. Data are presented from animals in which echocardiographic assessment was performed (*n* = 5 per group), as echocardiography was incorporated during the study as part of protocol refinement.

## Data Availability

The data sets generated during and/or analyzed during the current study are available from the corresponding author on reasonable request.

## Abbreviations

The following abbreviations are used in this manuscript:

HI	hypoxic–ischemic
ICAM-1	intercellular adhesion molecule-1
iNOS	inducible nitric oxide synthase
PPHN	persistent pulmonary hypertension of the newborn
H_2_	Hydrogen gas
HIE	Hypoxic–ischemic encephalopathy
OI	oxygen index
AaDO_2_	alveolar–arterial oxygen difference
FiO_2_	inspired oxygen concentration
SpO_2_	oxygen saturation
SV	Stroke volume
RVOT-VTI	right ventricular outflow tract velocity–time integral
CO	Cardiac output
